# Supplementary material for: Random intercept and linear mixed models including heteroscedasticity in a logarithmic scale: Correction terms and prediction in the original scale
Source: PLoS One. 2021 Apr 14;16(4):e0249910. doi: 10.1371/journal.pone.0249910 (PMC8046211; doi:10.1371/journal.pone.0249910)
Supplement: S1 File — (PDF) [file pone.0249910.s001.pdf]

S1 File
Random intercept model including heteroscedasticity in a logarithmic scale: correction terms and prediction in the original scale

Ricardo Ramírez-Aldana[1*],Lizbeth Naranjo[2]

1 Instituto Nacional de Geriatría, Ciudad de México, Mexico
2 Departamento de Matemáticas, Facultad de Ciencias, Universidad Nacional Autónoma de México, Ciudad de México, Mexico

* ricardoramirezaldana@gmail.com

Propositions

Proposition 1. *In the RIM with heteroscedasticity defined in (1), the variance associated with the distribution of the random effect* γ_i *conditional to the transformed response for the sample,* $\log(\mathbf{Y}) = (\log(\mathbf{Y}_1), \ldots, \log(\mathbf{Y}_m))'$*, with* $\mathbf{Y}_i = (Y_{i1}, \ldots, Y_{in_i})$ *for* $i = 1, \ldots, m$*, corresponds to*

$$Var(\gamma_i | \log(\mathbf{Y})) = \sigma^2_\gamma \left(1 - \frac{\sigma^2_\gamma}{\sigma^2_\gamma + \frac{\sigma^2}{\sum_{j=1}^{n_i} w_{ij}}} \right).$$

Proof. For this purpose, we will use the following known results of linear algebra and probability:

Result 1. *A block diagonal matrix* B *partitioned in* m *blocks, i.e.* $B = \text{diag}(B_1, B_2, \ldots, B_m)$*, satisfies that* $B^{-1} = \text{diag}(B_1^{-1}, B_2^{-1}, \ldots, B_m^{-1})$.

Result 2. *If* C *and* $C + D$ *are invertible matrices and* D *has rank 1, then* $(C + D)^{-1} = C^{-1} - \frac{1}{1+g} C^{-1} D C^{-1}$*, where* $g = \text{tr}(DC^{-1})$.

Result 3. *In a random vector having a multivariate Gaussian distribution, partitioned as follows:*

$$\begin{pmatrix} \mathbf{Y} \\ \mathbf{Z} \end{pmatrix} \sim N_p \left(\begin{pmatrix} \boldsymbol{\mu}_Y \\ \boldsymbol{\mu}_Z \end{pmatrix}, \begin{pmatrix} \Sigma_{YY} & \Sigma_{YZ} \\ \Sigma_{ZY} & \Sigma_{ZZ} \end{pmatrix} \right),$$

the conditional distribution of $\mathbf{Z}$ *given* $\mathbf{Y}$ *is a Gaussian distribution with expectation and variance given by* $\text{E}(\mathbf{Z}|\mathbf{Y}) = \boldsymbol{\mu}_Z + \Sigma_{ZY}\Sigma_{YY}^{-1}(\mathbf{y} - \boldsymbol{\mu}_Y)$ *and* $Var(\mathbf{Z}|\mathbf{Y}) = \Sigma_{ZZ} - \Sigma_{ZY}\Sigma_{YY}^{-1}\Sigma_{YZ}$.

First, we obtain $V = \text{Var}[\log(\mathbf{Y})]$, the variance and covariance matrix with dimension $n \times n$ associated with the transformed response. Matrix V is a block diagonal matrix, $V = \text{diag}(V_1, \ldots, V_m)$, according to the independence assumptions within and between the random effects and error terms. Under these assumptions, we observe that for a cluster i, $i = 1, ..., m$, $\text{Var}(\log(Y_{ij})) = \text{Var}(\mathbf{x}'_{ij}\boldsymbol{\beta} + \gamma_i + \epsilon_{ij}) = \sigma^2_\gamma + \sigma^2 w_{ij}^{-1}$, and $\text{Cov}(\log(Y_{ij}), \log(Y_{ik})) = \sigma^2_\gamma$; for $j \neq k$, hence

$$V_i = \begin{pmatrix} \sigma^2 w_{i1}^{-1} + \sigma_\gamma^2 & \sigma_\gamma^2 & \cdots & \sigma_\gamma^2 \\ \sigma_\gamma^2 & \sigma^2 w_{i2}^{-1} + \sigma_\gamma^2 & \cdots & \sigma_\gamma^2 \\ \vdots & \vdots & \ddots & \vdots \\ \sigma_\gamma^2 & \sigma_\gamma^2 & \cdots & \sigma^2 w_{in_i}^{-1} + \sigma_\gamma^2 \end{pmatrix}. \tag{1}$$

Using Result 3 for the conditional distribution $\gamma_i | \log(\mathbf{Y})$, we have that $\Sigma_{ZZ} = \sigma_\gamma^2$, $\Sigma_{ZY} = (\sigma_\gamma^2 \mathbf{1}'_{n_i} \ \mathbf{0}_{n-n_i})$, with $\mathbf{1}_{n_i}$ a n_i-dimensional vector containing only ones and similarly for the vector of zeroes $\mathbf{0}_{n-n_i}$, and $\Sigma_{YZ} = \Sigma'_{ZY}$, assuming that the elements in cluster i are presented first. Thus, for each cluster i, $i = 1, ..., m$,

$$\mathrm{Var}\left(\gamma_i | \log(\mathbf{Y})\right) = \sigma_\gamma^2 - (\sigma_\gamma^2 \mathbf{1}'_{n_i} \ \mathbf{0}_{n-n_i}) V^{-1} (\sigma_\gamma^2 \mathbf{1}'_{n_i} \ \mathbf{0}_{n-n_i})', \tag{2}$$

assuming also that the block for cluster i in V is presented first. Thus, the term $(\sigma_\gamma^2 \mathbf{1}'_{n_i} \ \mathbf{0}_{n-n_i}) V^{-1} (\sigma_\gamma^2 \mathbf{1}'_{n_i} \ \mathbf{0}_{n-n_i})'$, is equivalent to obtain the (i, i) element in

$$\mathrm{diag}\left(\sigma_\gamma^2 \mathbf{1}'_{n_1}, \sigma_\gamma^2 \mathbf{1}'_{n_2}, \ldots, \sigma_\gamma^2 \mathbf{1}'_{n_m}\right) V^{-1} \mathrm{diag}\left(\sigma_\gamma^2 \mathbf{1}'_{n_1}, \sigma_\gamma^2 \mathbf{1}'_{n_2}, \ldots, \sigma_\gamma^2 \mathbf{1}'_{n_m}\right)'.$$

According to (1), matrix V is a block diagonal matrix having m blocks, where its ith block, for $i = 1, \ldots, m$, corresponds to:

$$V_i = \sigma^2 \mathrm{diag}\left(w_{i1}^{-1}, w_{i2}^{-1}, \ldots, w_{in_i}^{-1}\right) + \sigma_\gamma^2 \mathbf{1}_{n_i} \mathbf{1}'_{n_i},$$

with $\mathbf{1}_{n_i} \mathbf{1}'_{n_i}$ a $n_i \times n_i$ matrix consisting of ones, thus it has a rank of one. In particular, we have that $V_i = C_i + D_i$, where $C_i = \sigma^2 \mathrm{diag}\left(w_{i1}^{-1}, w_{i2}^{-1}, \ldots, w_{in_i}^{-1}\right)$ and $D_i = \sigma_\gamma^2 \mathbf{1}_{n_i} \mathbf{1}'_{n_i}$. Additionally, by using Result 1, it is obtained that $C_i^{-1} = \frac{1}{\sigma^2} \mathrm{diag}\left(w_{i1}, w_{i2}, \ldots, w_{in_i}\right)$. Hence,

$$\begin{aligned} g_i &= \mathrm{tr}(D_i C_i^{-1}) \\ &= \mathrm{tr}\left(\sigma_\gamma^2 \mathbf{1}_{n_i} \mathbf{1}'_{n_i} \tfrac{1}{\sigma^2} \mathrm{diag}\left(w_{i1}, w_{i2}, \ldots, w_{in_i}\right)\right) \\ &= \mathrm{tr}\left(\frac{\sigma_\gamma^2}{\sigma^2} \begin{pmatrix} w_{i1} & w_{i2} & \cdots & w_{in_i} \\ w_{i1} & w_{i2} & \cdots & w_{in_i} \\ \vdots & \vdots & \ddots & \vdots \\ w_{i1} & w_{i2} & \cdots & w_{in_i} \end{pmatrix}\right) \\ &= \frac{\sigma_\gamma^2}{\sigma^2} \sum_{j=1}^{n_i} w_{ij}. \end{aligned}$$

On the other hand,

$$\begin{aligned} C_i^{-1} D_i C_i^{-1} &= \tfrac{1}{\sigma^2} \mathrm{diag}\left(w_{i1}, w_{i2}, \ldots, w_{in_i}\right) \sigma_\gamma^2 \mathbf{1}_{n_i} \mathbf{1}'_{n_i} \tfrac{1}{\sigma^2} \mathrm{diag}\left(w_{i1}, w_{i2}, \ldots, w_{in_i}\right) \\ &= \frac{\sigma_\gamma^2}{\sigma^4} \begin{pmatrix} w_{i1}^2 & w_{i1} w_{i2} & \cdots & w_{i1} w_{in_i} \\ w_{i1} w_{i2} & w_{i2}^2 & \cdots & w_{i2} w_{in_i} \\ \vdots & \vdots & \ddots & \vdots \\ w_{i1} w_{in_i} & w_{i2} w_{in_i} & \cdots & w_{in_i}^2 \end{pmatrix}. \end{aligned}$$

Hence, by using Result 2,

$$
\begin{aligned}
V_i^{-1} &= (C_i + D_i)^{-1} \\
&= \frac{1}{\sigma^2}\mathrm{diag}\left(w_{i1}, w_{i2}, \ldots, w_{in_i}\right) \\
&\quad - \frac{1}{1 + \frac{\sigma_\gamma^2}{\sigma^2}\sum_{j=1}^{n_i} w_{ij}} \frac{\sigma_\gamma^2}{\sigma^4} \begin{pmatrix} w_{i1}^2 & w_{i1}w_{i2} & \cdots & w_{i1}w_{in_i} \\ w_{i1}w_{i2} & w_{i2}^2 & \cdots & w_{i2}w_{in_i} \\ \vdots & \vdots & \ddots & \vdots \\ w_{i1}w_{in_i} & w_{i2}w_{in_i} & \cdots & w_{in_i}^2 \end{pmatrix} \\
&= \frac{1}{\sigma^2}\mathrm{diag}\left(w_{i1}, w_{i2}, \ldots, w_{in_i}\right) \\
&\quad - \frac{\sigma_\gamma^2}{\sigma^2(\sigma^2 + \sigma_\gamma^2\sum_{j=1}^{n_i} w_{ij})} \begin{pmatrix} w_{i1}^2 & w_{i1}w_{i2} & \cdots & w_{i1}w_{in_i} \\ w_{i1}w_{i2} & w_{i2}^2 & \cdots & w_{i2}w_{in_i} \\ \vdots & \vdots & \ddots & \vdots \\ w_{i1}w_{in_i} & w_{i2}w_{in_i} & \cdots & w_{in_i}^2 \end{pmatrix}.
\end{aligned}
$$

By using Result 1, we have that

$$
\begin{aligned}
\mathrm{diag}\left(\sigma_\gamma^2\mathbf{1}'_{n_1}, \sigma_\gamma^2\mathbf{1}'_{n_2}, \ldots, \sigma_\gamma^2\mathbf{1}'_{n_m}\right)V^{-1} &= \mathrm{diag}\left(\sigma_\gamma^2\mathbf{1}'_{n_1}, \sigma_\gamma^2\mathbf{1}'_{n_2}, \ldots, \sigma_\gamma^2\mathbf{1}'_{n_m}\right)\mathrm{diag}\left(V_1^{-1}, V_2^{-1}, \ldots, V_m^{-1}\right) \\
&= \mathrm{diag}\left(\sigma_\gamma^2\mathbf{1}'_{n_1}V_1^{-1}, \sigma_\gamma^2\mathbf{1}'_{n_2}V_2^{-1}, \ldots, \sigma_\gamma^2\mathbf{1}'_{n_m}V_m^{-1}\right).
\end{aligned}
$$

The ith block, given by $\sigma_\gamma^2\mathbf{1}'_{n_i}V_i^{-1}$, corresponds to the row vector

$$
\sigma_\gamma^2\left(\frac{w_{i1}}{\sigma^2} - \frac{\sigma_\gamma^2\sum_{j=1}^{n_i} w_{i1}w_{ij}}{\sigma^2(\sigma^2 + \sigma_\gamma^2\sum_{j=1}^{n_i} w_{ij})}, \ldots, \frac{w_{in_i}}{\sigma^2} - \frac{\sigma_\gamma^2\sum_{j=1}^{n_i} w_{in_i}w_{ij}}{\sigma^2(\sigma^2 + \sigma_\gamma^2\sum_{j=1}^{n_i} w_{ij})}\right).
$$

Matrix $\mathrm{diag}\left(\sigma_\gamma^2\mathbf{1}'_{n_1}, \sigma_\gamma^2\mathbf{1}'_{n_2}, \ldots, \sigma_\gamma^2\mathbf{1}'_{n_m}\right)V^{-1}\mathrm{diag}\left(\sigma_\gamma^2\mathbf{1}'_{n_1}, \sigma_\gamma^2\mathbf{1}'_{n_2}, \ldots, \sigma_\gamma^2\mathbf{1}'_{n_m}\right)'$, which is clearly a diagonal matrix whose dimension is $m \times m$, has as ith diagonal element $\sigma_\gamma^2\mathbf{1}'_{n_i}V_i^{-1}\sigma_\gamma^2\mathbf{1}_{n_i}$, corresponding to

$$
\begin{aligned}
\sigma_\gamma^4\mathbf{1}'_{n_i}V_i^{-1}\mathbf{1}_{n_i} &= \sigma_\gamma^4\left(\frac{1}{\sigma^2}\sum_{j=1}^{n_i} w_{ij} - \frac{\sigma_\gamma^2}{\sigma^2(\sigma^2 + \sigma_\gamma^2\sum_{j=1}^{n_i} w_{ij})}\sum_{k=1}^{n_i}\sum_{j=1}^{n_i} w_{ik}w_{ij}\right) \\
&= \sigma_\gamma^4\left(\frac{\sigma^2\sum_{j=1}^{n_i} w_{ij} + \sigma_\gamma^2(\sum_{j=1}^{n_i} w_{ij})^2 - \sigma_\gamma^2(\sum_{j=1}^{n_i} w_{ij})^2}{\sigma^2(\sigma^2 + \sigma_\gamma^2\sum_{j=1}^{n_i} w_{ij})}\right) \\
&= \sigma_\gamma^2\left(\frac{\sigma_\gamma^2}{\sigma_\gamma^2 + \frac{\sigma^2}{\sum_{j=1}^{n_i} w_{ij}}}\right).
\end{aligned}
$$

Thus, according to (2), $\mathrm{Var}(\gamma_i|\log(\mathbf{Y}))$, corresponds to

$$
\mathrm{Var}(\gamma_i|\log(\mathbf{Y})) = \sigma_\gamma^2\left(1 - \frac{\sigma_\gamma^2}{\sigma_\gamma^2 + \frac{\sigma^2}{\sum_{j=1}^{n_i} w_{ij}}}\right).
$$

As a particular case, under homoscedasticity, $w_{ij} = 1$, for all $i = 1, \ldots, m$ and $j = 1, \ldots, n_i$, and the desired element corresponds to:

$$
\mathrm{Var}(\gamma_i|\log(\mathbf{Y})) = \sigma_\gamma^2\left(1 - \frac{\sigma_\gamma^2}{\sigma_\gamma^2 + \frac{\sigma^2}{n_i}}\right).
$$

□

Proposition 2. *In the LMM with heteroscedasticity defined in (14), the variance associated with the distribution of the vector of random effects* $\boldsymbol{\gamma}_i$ *conditional to the transformed response for the sample,* $\log(\mathbf{Y}) = (\log(\mathbf{Y}_1), \ldots, \log(\mathbf{Y}_m))'$*, with* $\mathbf{Y}_i = (Y_{i1}, \ldots, Y_{in_i})$ *for* $i = 1, \ldots, m$*, corresponds to*

$$Var(\boldsymbol{\gamma}_i | \log(\mathbf{Y})) = D - DU_i'(U_i D U_i' + \Sigma_i)^{-1} U_i D.$$

Proof. From the matrix form associated with the model

$$\log(\mathbf{Y}) = \mathbf{X}\boldsymbol{\beta} + U\boldsymbol{\gamma} + \boldsymbol{\epsilon},$$

and using the model assumptions, the variance and covariance matrix associated with the transformed response V is $V = \mathrm{Var}(\log(\mathbf{Y})) = UGU' + R$ with dimension $n \times n$, and the joint distribution associated with $\log(\mathbf{Y})$ and $\boldsymbol{\gamma}$ corresponds to:

$$\begin{pmatrix} \log(\mathbf{Y}) \\ \boldsymbol{\gamma} \end{pmatrix} \sim N_{n+m}\left(\begin{pmatrix} \mathbf{X}\boldsymbol{\beta} \\ \mathbf{0}_{m\times 1} \end{pmatrix}, \begin{pmatrix} V & UG \\ GU' & G \end{pmatrix} \right). \tag{3}$$

Thus, applying in (3) Result 3 concerning the conditional distribution and partition of multivariate normal distributions, we have that $\mathrm{Var}(\boldsymbol{\gamma} | \log(\mathbf{Y})) = G - GU'V^{-1}UG$. The variance and covariance matrix associated with cluster i, $\mathrm{Var}(\boldsymbol{\gamma}_i | \log(\mathbf{Y}))$, corresponds to the ith block in $\mathrm{Var}(\boldsymbol{\gamma} | \log(\mathbf{Y}))$. Using that $V = UGU' + R$ and that G, U, and R are all block diagonal matrices, it is easy to derive that the ith block of $G - GU'V^{-1}UG$ corresponds to $D - DU_i'(U_i D U_i' + \Sigma_i)^{-1} U_i D$, and consequently

$$\mathrm{Var}(\boldsymbol{\gamma}_i | \log(\mathbf{Y})) = D - DU_i'(U_i D U_i' + \Sigma_i)^{-1} U_i D.$$

□

Mimic simulation example

A sample of $n = 2000$ subjects were simulated by the following process.The covariates $\mathbf{x}_{ij}'$ were the same as in the income for elderly people data in the $m = 32$ states in Mexico. The selection of the values of the parameters were close to the estimates obtained in the analysis of the income for elderly people data, see Section 4.2.

Linear predictors were computed by using the following regression parameter values $\boldsymbol{\beta} = (9.128, -0.176, 0.042, -0.064, -0.069,\ 0.047, 0.171, 0.347, 0.760, 0.222,$ $0.443, 0.625, 0.685,\ -0.057, -0.160,\ -0.465, -0.120, -0.415, -0.452, -0.407,$ $-0.075, -0.201, 0.023, -0.106,\ 0.014,\ -0.130, -0.089, -0.092, -0.136, -0.115,$ $-0.014, -0.005, 0.096, 0.074,\ 0.144, 0.085,\ 0.183)$, and the intercept random effects γ_i and the random errors ε_{ij} were simulated by using the standard deviations $\sigma_\gamma = \{0.079, 0.16, 0.32, 0.64, 1.28\}$ and $\sigma = \{0.15, 0.3, 0.594, 0.9, 1.2\}$, respectively. Since in the original data there were not weights, in order to introduce heteroscedasticity we used two types of weights: (1) the size of each cluster, thus subjects in the same cluster have the same weight, assuming that larger states should have less variability and the smaller states greater variability in their income; and (2) proportional to the total number of light bulbs, where more number of light bulbs are related with a better socioeconomic level, being the variability of the income lower with more number of light bulbs and viceversa. The true responses $\log(Y_{ij})$'s were generated by using Equation (1), and then, the responses Y_{ij}'s were obtained applying the exponential transformation. Note that the way these data were generated satisfies the assumptions defined for the RIM. From the true model, 100 datasets with true responses were simulated.

S1 Table. Summary of the MSE for the mimic simulation example.

Weights	σ	σ_γ	MSE_{naive} mean (sd)	$MSE_{(5)}$ mean (sd)	$MSE_{(9)}$ mean (sd)	$MSE_{(11)}$ mean (sd)	$MSE_{(13)}$ mean (sd)
(1)	0.15	0.079	183.0 (5.9)	797.6 (104.2)	183.0 (5.9)	183.0 (5.9)	183.0 (5.9)
(1)	0.15	0.16	185.9 (7.6)	1552.4 (235.2)	185.9 (7.6)	185.9 (7.6)	185.9 (7.6)
(1)	0.15	0.32	201.7 (16.2)	3383.0 (607.6)	201.7 (16.2)	201.7 (16.2)	201.7 (16.2)
(1)	0.15	0.64	262.9 (41.0)	7728.6 (2038.7)	262.9 (41.0)	262.9 (41.0)	262.9 (41.0)
(1)	0.15	1.28	753.6 (350.0)	35308.6 (19790.8)	753.8 (350.0)	753.8 (350.0)	753.7 (350.0)
(1)	0.3	0.079	366.1 (10.7)	845.4 (101.0)	366.0 (10.7)	366.0 (10.7)	366.1 (10.7)
(1)	0.3	0.16	373.0 (15.4)	1623.9 (254.1)	372.9 (15.4)	372.9 (15.4)	372.9 (15.4)
(1)	0.3	0.32	404.6 (33.0)	3332.4 (648.9)	404.5 (33.0)	404.5 (33.0)	404.5 (33.0)
(1)	0.3	0.64	540.4 (104.2)	8066.4 (2778.6)	540.2 (104.1)	540.2 (104.1)	540.2 (104.0)
(1)	0.3	1.28	1442.5 (731.3)	31711.0 (18101.3)	1442.3 (730.8)	1442.3 (730.8)	1442.2 (730.9)
(1)	0.594	0.079	725.3 (22.4)	1053.2 (84.1)	724.6 (22.3)	724.6 (22.3)	724.7 (22.3)
(1)	0.594	0.16	741.1 (31.2)	1746 (225.7)	740.5 (31.1)	740.5 (31.1)	740.6 (31.0)
(1)	0.594	0.32	796.7 (67.6)	3432.5 (696.9)	796.0 (67.5)	796.0 (67.5)	796.0 (67.5)
(1)	0.594	0.64	1058.5 (180.3)	7927.4 (2311.2)	1057.6 (180.1)	1057.5 (180.1)	1057.6 (180.1)
(1)	0.594	1.28	2994.2 (2264.1)	34012.2 (27261.6)	2991.4 (2262.3)	2991.3 (2262.3)	2991.9 (2262.5)
(1)	0.9	0.079	1110.8 (35.1)	1360.0 (74.4)	1108.7 (35.2)	1108.7 (35.2)	1108.9 (35.1)
(1)	0.9	0.16	1134.8 (47.2)	1928.9 (208.5)	1132.5 (47.0)	1132.5 (47.0)	1132.7 (47.0)
(1)	0.9	0.32	1229.4 (79.6)	3651.3 (575.1)	1227.1 (79.4)	1227.1 (79.3)	1227.3 (79.4)
(1)	0.9	0.64	1667.9 (315.4)	8556.6 (2549.2)	1665.5 (315.2)	1665.5 (315.2)	1665.7 (315.0)
(1)	0.9	1.28	4596.0 (2544.9)	35743.4 (25158.9)	4592.5 (2549.9)	4592.6 (2550.0)	4593.8 (2551.3)
(1)	1.2	0.079	1495.4 (49.6)	1680.7 (68.5)	1490.5 (49.1)	1490.4 (49.1)	1490.7 (49.1)
(1)	1.2	0.16	1523.1 (69.0)	2180.2 (186.1)	1518.1 (68.5)	1518.1 (68.5)	1518.5 (68.6)
(1)	1.2	0.32	1634.4 (107.4)	3713.1 (592.2)	1629.5 (106.4)	1629.5 (106.4)	1629.9 (106.5)
(1)	1.2	0.64	2213.8 (356.5)	8580.3 (2442.9)	2208.0 (356.4)	2208.0 (356.4)	2208.6 (356.4)
(1)	1.2	1.28	6852.0 (5789.4)	38593.9 (36263.5)	6840.1 (5851.4)	6840.2 (5852.4)	6844.1 (5869.6)
(2)	0.15	0.079	540.1 (12.1)	928.9 (88.2)	539.9 (12.1)	539.9 (12.1)	539.9 (12.1)
(2)	0.15	0.16	550.2 (21.6)	1673.2 (237.0)	549.9 (21.6)	549.9 (21.6)	550.0 (21.6)
(2)	0.15	0.32	592.8 (38.2)	3306.8 (562.7)	592.5 (38.2)	592.5 (38.2)	592.6 (38.2)
(2)	0.15	0.64	776.6 (115.0)	7822.4 (2068.5)	776.2 (115.0)	776.2 (115.0)	776.3 (115.0)
(2)	0.15	1.28	2290.2 (1369.8)	35322.8 (24328.5)	2288.1 (1367.9)	2288.1 (1367.9)	2289.4 (1369.4)
(2)	0.3	0.079	1097.5 (30.0)	1348.8 (60.9)	1095.2 (29.4)	1095.2 (29.4)	1095.9 (29.8)
(2)	0.3	0.16	1119.9 (38.8)	1937.0 (200.3)	1117.4 (38.5)	1117.4 (38.5)	1118.2 (38.7)
(2)	0.3	0.32	1199.7 (88.7)	3528.1 (538.1)	1196.8 (88.1)	1196.8 (88.1)	1197.8 (88.4)
(2)	0.3	0.64	1600.2 (294.5)	8191.0 (2491.8)	1597.0 (293.9)	1597.0 (293.9)	1597.6 (293.5)
(2)	0.3	1.28	4594.0 (2427.4)	35429.0 (20796.2)	4591.1 (2433.0)	4591.2 (2433.1)	4591.3 (2431.5)
(2)	0.594	0.079	2264.4 (81.6)	2387.4 (86.0)	2245.5 (78.5)	2245.5 (78.5)	2251.4 (80.5)
(2)	0.594	0.16	2315.2 (96.2)	2805.6 (187.1)	2295.2 (93.9)	2295.2 (93.9)	2301.5 (95.4)
(2)	0.594	0.32	2506.9 (206.4)	4311.0 (683.5)	2484.7 (200.4)	2484.7 (200.3)	2491.3 (203.9)
(2)	0.594	0.64	3378.7 (549.0)	8982.0 (2217.6)	3353.6 (543.2)	3353.6 (543.2)	3359.4 (546.8)
(2)	0.594	1.28	8994.3 (4847.5)	34063.3 (21229.0)	8909.1 (4786.8)	8908.9 (4786.4)	8934.1 (4792.5)
(2)	0.9	0.079	3758.2 (255.5)	3790.5 (242.0)	3693.5 (241.1)	3693.5 (241.0)	3716.7 (253.7)
(2)	0.9	0.16	3790.1 (197.0)	4101.0 (220.4)	3726.8 (182.6)	3726.8 (182.5)	3745.8 (193.7)
(2)	0.9	0.32	4062.5 (417.5)	5252.5 (648.2)	3992.8 (406.6)	3992.8 (406.6)	4014.3 (415.3)
(2)	0.9	0.64	5618.2 (1059.0)	10597.6 (2671.2)	5525.8 (1028.2)	5525.7 (1028.1)	5549.6 (1042.8)
(2)	0.9	1.28	14806.0 (8984.8)	38744.9 (26806.1)	14604.4 (8883.0)	14605.0 (8883.7)	14661.7 (8927.3)
(2)	1.2	0.079	5546.3 (647.1)	5472.5 (622.3)	5412.8 (629.8)	5412.8 (629.7)	5453.8 (648.7)
(2)	1.2	0.16	5773.7 (699.9)	5896.0 (679.3)	5615.0 (670.1)	5615.0 (669.9)	5672.1 (699.8)
(2)	1.2	0.32	6289.4 (1296.7)	7195.0 (1295.5)	6122.4 (1257.0)	6122.4 (1256.9)	6182.5 (1301.5)
(2)	1.2	0.64	8369.8 (1920.9)	12353.7 (2878.3)	8161.0 (1832.4)	8161.1 (1832.2)	8225.9 (1900.0)
(2)	1.2	1.28	23639.7 (17407.2)	47081.2 (37670.3)	23141.8 (17392.3)	23143.1 (17395.0)	23264.0 (17244.1)

(1) Size of each cluster. (2) Total number of light bulbs.
