# Supplementary material for: Random intercept and linear mixed models including heteroscedasticity in a logarithmic scale: Correction terms and prediction in the original scale
Source: PLoS One. 2021 Apr 14;16(4):e0249910. doi: 10.1371/journal.pone.0249910 (PMC8046211; doi:10.1371/journal.pone.0249910)
Supplement: Attachment — Submitted filename: Response to Reviewers_08122020.pdf [file pone.0249910.s007.pdf]

PONE-D-20-31434

PLOS ONE

Random intercept model including heteroscedasticity in a logarithmic scale: correction terms and prediction in the original scale

Ricardo Ramírez-Aldana, Lizbeth Naranjo

Academic Editor

Dear Dr. Ramírez-Aldana,

Thank you for submitting your manuscript to PLOS ONE. One of the referees have recommended that you make amendments to your article. We therefore invite you to submit a revised version of the manuscript that addresses the points raised during the review process.

Please submit your revised manuscript by Dec 25 2020 11:59PM. If you will need more time than this to complete your revisions, please reply to this message or contact the journal office at `plosone@plos.org`*. When you're ready to submit your revision, log on to* `https://www.editorialmanager.com/pone/` *and select the 'Submissions Needing Revision' folder to locate your manuscript file.*

Please include the following items when submitting your revised manuscript:

- *A rebuttal letter that responds to each point raised by the academic editor and reviewer(s). You should upload this letter as a separate file labeled 'Response to Reviewers'.*
- *A marked-up copy of your manuscript that highlights changes made to the original version. You should upload this as a separate file labeled 'Revised Manuscript with Track Changes'.*
- *An unmarked version of your revised paper without tracked changes. You should upload this as a separate file labeled 'Manuscript'.*

If you would like to make changes to your financial disclosure, please include your updated statement in your cover letter. Guidelines for resubmitting your figure files are available below the reviewer comments at the end of this letter.

If applicable, we recommend that you deposit your laboratory protocols in protocols.io to enhance the reproducibility of your results. Protocols.io assigns your protocol its own identifier (DOI) so that it can be cited independently in the future. For instructions see: http://journals.plos.org/plosone/s/submission-guidelines#loc-laboratory-protocols

We look forward to receiving your revised manuscript.

Kind regards,

Ivan Kryven
Academic Editor
PLOS ONE

Journal Requirements:

When submitting your revision, we need you to address these additional requirements.

1. Please ensure that your manuscript meets PLOS ONE's style requirements, including those for file naming. The PLOS ONE style templates can be found at

https://journals.plos.org/plosone/s/file?id=wjVg/PLOSOne_formatting_sample_main_body.pdf *and*

https://journals.plos.org/plosone/s/file?id=ba62/PLOSOne_formatting_sample_title_authors_affiliations.pdf

2. We suggest you thoroughly copyedit your manuscript for language usage, spelling, and grammar. If you do not know anyone who can help you do this, you may wish to consider employing a professional scientific editing service.

*Whilst you may use any professional scientific editing service of your choice, PLOS has partnered with both American Journal Experts (AJE) and Editage to provide discounted services to PLOS authors. Both organizations have experience helping authors meet PLOS guidelines and can provide language editing, translation, manuscript formatting, and figure formatting to ensure your manuscript meets our submission guidelines. To take advantage of our partnership with AJE, visit the AJE website (*http://learn.aje.com/plos/*) for a 15% discount off AJE services. To take advantage of our partnership with Editage,*

visit the Editage website (`www.editage.com`) and enter referral code PLOSEDIT for a 15% discount off Editage services. If the PLOS editorial team finds any language issues in text that either AJE or Editage has edited, the service provider will re-edit the text for free.

Upon resubmission, please provide the following:

- *The name of the colleague or the details of the professional service that edited your manuscript*
- *A copy of your manuscript showing your changes by either highlighting them or using track changes (uploaded as a *supporting information* file)* A clean copy of the edited manuscript (uploaded as the new *manuscript* file)
- *A clean copy of the edited manuscript (uploaded as the new *manuscript* file)*

3. Please include captions for your Supporting Information files at the end of your manuscript, and update any in-text citations to match accordingly. Please see our Supporting Information guidelines for more information: `http://journals.plos.org/plosone/s/supporting-information`.

Response:

Thank you very much for the revision.

We have modified the paper attending to the comments and suggestions raised by the two reviewers. A point-by-point response is provided here. The modifications have been marked in red color in the new version of the paper.

Reviewers' comments:

Reviewer's Responses to Questions

Comments to the Author

---

1. Is the manuscript technically sound, and do the data support the conclusions?

The manuscript must describe a technically sound piece of scientific research with data that supports the conclusions. Experiments must have been conducted rigorously, with appropriate controls, replication, and sample sizes. The conclusions must be drawn appropriately based on the data presented.

Reviewer #1: Yes

Reviewer #2: Yes

---

2. Has the statistical analysis been performed appropriately and rigorously?

Reviewer #1: Yes

Reviewer #2: Yes

---

3. Have the authors made all data underlying the findings in their manuscript fully available?

The PLOS Data policy requires authors to make all data underlying the findings described in their manuscript fully available without restriction, with rare exception (please refer to the Data Availability Statement in the manuscript PDF file). The data should be provided as part of the manuscript or its supporting information, or deposited to a public repository. For example, in addition to summary statistics, the data points behind means, medians and variance measures should be available. If there are restrictions on publicly sharing data—e.g. participant privacy or use of data from a third party—those must be specified.

Reviewer #1: No

Response

The link associated with the data is provided in the reference (last reference of the manuscript) concerning the National Household Income and Expenditure

Survey 2016 (ENIGH 2016). The R code, included as Supplementary material, concerning the example provides once again the link and includes detailed explanations of how to use and transform the data available there to generate all data used in the manuscript.

Reviewer #2: Yes

---

4. Is the manuscript presented in an intelligible fashion and written in standard English?

PLOS ONE does not copyedit accepted manuscripts, so the language in submitted articles must be clear, correct, and unambiguous. Any typographical or grammatical errors should be corrected at revision, so please note any specific errors here.

Reviewer #1: Yes

Reviewer #2: Yes

---

5. Review Comments to the Author

Please use the space provided to explain your answers to the questions above. You may also include additional comments for the author, including concerns about dual publication, research ethics, or publication ethics. (Please upload your review as an attachment if it exceeds 20,000 characters)

Reviewer #1:

This paper proposes and evaluates correction methods for analysis using log-transformed data to fit a linear mixed model and using the fit to obtain predictions on the original scale.

Response

Thank you very much for the revision.

We really appreciate your time in reviewing the paper.

We have modified the paper attending to the comments and suggestions raised by you. A point-by-point response is provided here. The modifications have been marked in red color in the new version of the paper.

(1) *The correction follows from the property of the log-normal distribution. If mu and var are the mean and variance on the log-scale than exp(mu + var/2) is the mean on the original scale. This is well-known, so the claim made in the paper that the correction is proposed "for the first time" is probably overstating the case.*

Response

What we meant is that it is the first time as far as the authors know that the corrections terms have been obtained for linear mixed models (LMM), though the necessity of correction terms it is well known and used in linear regression models (L-82, previous version). We eliminated the words "for the first time" to not give the impression that we are overstating our results. As the reviewer stated, it is well known which one is the expression corresponding to the mean on the original scale of a log normal distribution and that it depends on a variance term. In fact, in our results we also saw that the correction terms depend on some type of estimated variance as well. However, we think that one of our contributions is that we identified that it was preferable to use a conditional distribution for a LMM, though the mentioned property concerning log normal distributions is used, and additionally we provided a closed and easy to use expression for the correction terms, at least for random intercept models (RIM).

(2) *That being said, the authors have a particular application. My feeling is that the whole*

story line should be more focused on the application case. Most importantly, I am missing a clear statement what exactly is to be estimated in the context of the example. Without that context, it is cumbersome to follow all the equations. Not because of the equations, but because it is unclear what we are estimating!

Response

We have added the application as a motivation example presented in the Introduction, including how the notation used in the manuscript can be seen in terms of the example (L-86). This example is followed through out the manuscript explaining what is introduced in each section (L-128, L-165). In this way, we clarify that what we want to estimate is income including a random effect for cluster (state), which is fitted in the logarithmic scale, but that there are other models, for instance a GLMM with gamma distribution, that also make sense for this kind of data and that they are fitted for comparison purposes. Later, the example is fully introduced in this revised version (L-351) in which we modified some lines (L-351, L-360, L-378, L-388, L-401, and L-406) to reflect what was said when the example was introduced before. We think that now is clearer the aim and the interpretation of the results and why we compare our results with specific models.

(3) *While I think it is important to focus more clearly on the example, I also think the scope needs to be broadened a bit for the benefit of readers using mixed models but not necessarily the one considered by the authors. The corrections are pretty straight-forward and applicable with other mixed models. All that's required is to work out the relevant marginal variance to use in the correction.*

Response

We had tried to keep the manuscript a little simpler, including only the RIM corrections since they have a closed formula depending only on scalars. However, now we have added a new section concerning linear mixed models (LMM) in general, being the results obtained for the RIM a particular case (L-413). In this case, the correction terms associated with the random effects have also an associated closed formula, but now in terms of matrices, which can be a little harder to implement. The process to obtain them was analogous to the one used for the RIM, thus, many of the results already introduced were used for this process. We have changed the title of the manuscript to identify that our approach is generalized to any LMM and modified some conclusions accordingly (L-520).

(4) *Model (2) only has a single subscript "i", whereas equation (4) has two subscripts "ij". It is not clear what these subscripts refer to and why they are changing in this way. Again, providing the specific context of your example may help here.*

Response

In section 2, we introduce the "correction term associated with a linear regression model in a logarithmic scale", and then equation (2), $\log(Y_i) = \mathbf{x}_i'\boldsymbol{\beta} + u_i$, refers to a linear regression model. On the other hand, in section 3 we analyze the "correction terms in a RIM with heteroscedasticity and a logarithmic scale", where from the random intercept model (RIM) in equation (1), $\log(Y_{ij}) = \mathbf{x}_{ij}'\boldsymbol{\beta} + \gamma_i + \epsilon_{ij}$, or equivalently $Y_{ij} = \exp\left(\mathbf{x}_{ij}'\boldsymbol{\beta} + \gamma_i + \epsilon_{ij}\right)$, we obtained the equation (4), $\mathrm{E}\left[Y_{ij}\right] = \exp\left(\mathbf{x}_{ij}'\boldsymbol{\beta}\right) \mathrm{E}\left[\exp\left(\gamma_i\right)\right] \mathrm{E}\left[\exp\left(\epsilon_{ij}\right)\right]$.

In simple words, two indexes were used to consider the nesting of individuals j in a cluster i, which makes sense when we are considering linear mixed models, and obviously a RIM. On the other hand, a single index was used to indicate a linear regression model (in a logarithmic scale) without random effects, being unnecessary the second index. We added into the manuscript in terms of the motivation example what this change in indexes means and explicitly explain why in only section 2 a single index is used (L-128).

(5) *In line 169, I am not sure why there is a need to switch to matrix notation. What does this really help? I am also not sure expressions like in eq. (12) are correct, where you are mixing matrix and scalar expressions.*

Response

Matrix notation in line 169 is used because it makes the calculations easier to obtain, being able to derive the closed formula associated with the correction terms. We have reviewed the calculations necessary to obtain (12), and they all are correct. The details are given in the Proposition provided in the Supplementary Material. Additionally, in this revised version, we included a section concerning the generalization for LMM, and all the matricial algebra and results introduced for the RIM were used there as well. We added in L-202 an explanation about these matters.

(6) *Line 219: What is the rationale underlying the smearing estimator, and what is it's*

advantage over the plug-in estimator based on what I said in (1)? Some explanation in the paper would be useful.

Response

In the section corresponding to corrections for the linear regression we already introduced the smearing estimator, stating that it is non-parametric. We have added a short explanation about what the smearing estimator is in terms of our corrections (L-254). We did not add more details concerning all properties of the estimator since they are extensively explained in other works, for instance in Duan (1983).

(7) *Line 267: It is important to tell readers how you simulated the data and why.*

Response

The data sets were generated with the specifications in the paragraphs above line 267 (previous version of the manuscript), that is, those explained at the beginning of section 4. We have clarified it since it seemed they were paragraphs apart (L-305). On the other hand, we included as part of the introduction a little more about the reason why and what we are simulating (L-107).

(8) *Line 274: Why did you even consider fitting this GLMM? It's not the model used to simulate the data, is it? Conversely, if it is assumed the GLMM generated the data, why use any other approach for analysis? Clearly, a rationale for using these alternate models is missing.*

Response

In a first type of simulations, the response is simulated using the log-transformed response and a LMM, thus assuming normality. Here, the GLMM with a gamma distribution was fitted to the data because it is an alternative to model positive skewed variables, as income or costs, avoiding transformations in the linear model and allowing to fit models under different distributional assumptions. For instance, Malehi et al. (2015) simulated data with different skewness to compare linear models assuming log-normal, gamma, or Weibull distributions (the latter two through GLM), among other models, and to identify

which one was better to model health care cost data. We have added this justification for using the GLMM in the manuscript in terms of the motivation example (L-102), as explained in Answer **(2)**, and some comments to clarify this in terms of the simulation as well (L-314). On the other hand, the second type of simulations are based on the GLMM with a gamma distribution.

(9) *Line 281: On a similar note, it is not clear to me why data were generated from a GLMM in a second simulation. I think you need a clear justification and story line for this choice.*

Response

We used a GLMM with a gamma distribution and a logarithmic link to simulate the data in order to see how our approach worked even when the true distribution associated with some data was not Gaussian, but it was one of the ones used in skewed positive data avoiding transforming the data as discussed in Answer **(8)**. We identified that in general our approach still worked as discussed in the Conclusions (L-375, previous version). Additionally, the comparison also makes sense since for particular values assigned to the shape and scale parameters, the behavior of the distribution can be similar to the one observed for a LMM in a logarithmic scale. We have added some comments to clarify this (L-330), not only in the Conclusions.

(10) *The manuscript file did not contain and of the diagnostic residual plots, so I was not able to convince myself that the log-normality assumption was satisfied. How do you actually check this when the data is heteroscedastic, as is the case under your model?*

Response

In theory, a random variable Y is distributed Log-Normal with parameters μ and σ^2 if $\log(Y)$ is distributed $Normal(\mu, \sigma^2)$. In figure 3, which corresponds to the application example, we included diagnostic residual plots: histogram, and qqplots associated with the error terms and random effects, to show that the normality assumption is satisfied for the LMM in the logarithmic scale (using $\log(Y)$).

In the application, we showed that residuals satisfy the log-normality assumption in a

homoscedastic framework. However, in the general case, the linear mixed model (LMM) is: $\log(Y_{ij}) = \mathrm{x}'_{ij}\boldsymbol{\beta} + \gamma_i + \varepsilon_{ij}$. The random error is ε_{ij}, and since heteroscedasticity is assummed $\varepsilon_{ij} \sim N(0, \sigma^2 w_{ij}^{-1})$ i.i.d., where w_{ij}^{-1} is a known number that allows different variability between observations and clusters. In the case of residuals, we need to show that residuals defined as $e_{ij} = \log(Y_{ij}) - \hat{\mathrm{E}}[\log(Y_{ij})]$ are normal distributed, and they are estimations of the errors, i.e. $e_{ij} = \hat{\varepsilon}_{ij}$. In the case of heteroscedasticity, as far as we know, there are not diagnostic tests specific when the data is heteroscedastic. However, we could use that $\varepsilon_{ij} w_{ij}^{1/2} \sim N(0, \sigma^2)$ to see that the model is adequate in terms of the distribution and the inclusion of heteroscedasticity terms.

Here we have included some plots to show the residuals in the heteroscedastic case. We have used one of the datasets having heteroscedasticity simulated in the fourth section, we have estimated the parameters and computed the residuals. We have plotted the residuals considering the weights; i.e. $e_{ij} w_{ij}^{1/2}$, and the corresponding qqplot and the log-normality assumption can be verified.

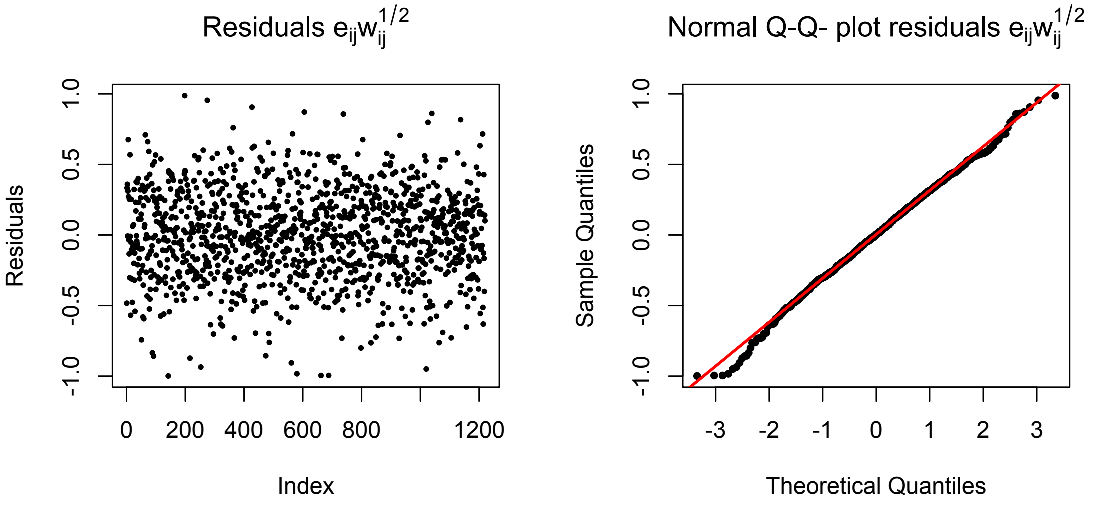

(11) *And now the obvious question: what to do if you are not so lucky that the log-transformation works well? You mention Box-Cox transformation in the discussion. What if I need to use* $x^{0.3}$*, say? How does your approach generalize? If you can cover that more general case, and also tell people what to do in case their LMM looks different than yours,*

you can vastly broaden the scope and impact of your paper. As it stands, it's a rather narrow case study.

Response

We have generalized the correction terms to any LMM and we outline the process when a different transformation is used. However, for the later case, we did not find an exact (matricial or not) solution, simulations should be used for this process (L-465). The conclusions were modified to include these results (L-523).

References

[1] Duan N. Smearing Estimate: A Nonparametric Retransformation Method. Journal of the American Statistical Association. 1983;78(383):605–610.

[2] Instituto Nacional de Estadística y Geografía (INEGI). Encuesta Nacional de Ingresos y Gastos de los Hogares 2016, ENIGH. Mexico: Instituto Nacional de Estadística y Geografía; 2016. Available from: `https://www.inegi.org.mx/programas/enigh/nc/2016/`.

[3] Malehi AS, Pourmotahari F, Angali KA. Statistical models for the analysis of skewed healthcare cost data: a simulation study Health Economics Review. 2015;5(1):11.

Reviewer #2:

Authors have proposed correction terms to predict the response in the original scale, which are easy to calculate and implement, for a random intercept model (RIM) with or without heteroscedasticity with response in a logarithmic scale. Different estimators of the predicted response are given (some of them already present in the literature) In addition, simulations and a real data set are presented in the paper to show the importance of using the correction terms to obtain more accurate predictions.

I have no comment whatsoever. Overall the manuscript is well written and organized. I enjoyed reading the paper and we can say that this paper contributes to the growing literature on statistical modeling.

Response

Thank you very much for the revision.

We really appreaciate your time in reviewing the paper.

---

6. PLOS authors have the option to publish the peer review history of their article (what does this mean?). If published, this will include your full peer review and any attached files.

If you choose "no", your identity will remain anonymous but your review may still be made public.

Do you want your identity to be public for this peer review? For information about this choice, including consent withdrawal, please see our Privacy Policy.

Reviewer #1: No

Reviewer #2: No

[NOTE: If reviewer comments were submitted as an attachment file, they will be attached to this email and accessible via the submission site. Please log into your account, locate the manuscript record, and check for the action link "View Attachments". If this link does not appear, there are no attachment files.]

While revising your submission, please upload your figure files to the Preflight Analysis and Conversion Engine (PACE) digital diagnostic tool, https://pacev2.apexcovantage.com/. PACE helps ensure that figures meet PLOS requirements. To use PACE, you must first register as a user. Registration is free. Then, login and navigate to the UPLOAD tab, where you will find detailed instructions on how to use the tool. If you encounter any issues or have any questions when using PACE, please email PLOS at figures@plos.org. Please note that Supporting Information files do not need this step.
