# Supplementary material for: Random intercept and linear mixed models including heteroscedasticity in a logarithmic scale: Correction terms and prediction in the original scale
Source: PLoS One. 2021 Apr 14;16(4):e0249910. doi: 10.1371/journal.pone.0249910 (PMC8046211; doi:10.1371/journal.pone.0249910)
Supplement: Attachment — Submitted filename: Response to Reviewers_27012021.pdf [file pone.0249910.s008.pdf]

PONE-D-20-31434R1

PLOS ONE

Random intercept model including heteroscedasticity in a logarithmic scale: correction terms and prediction in the original scale

Ricardo Ramírez-Aldana, Lizbeth Naranjo

Academic Editor

Dear Dr. Ramírez-Aldana,

Thank you for revising your manuscript. As you can see Reviewer 1 still has several comments and we therefore invite you to prepare a new version of the manuscript addressing Reviewer's feedback. Following on Reviewer's advise, please condense the description of the key steps of the approach and include more elaborated discussion of examples and extensions.

Please submit your revised manuscript by Feb 06 2021 11:59PM. If you will need more time than this to complete your revisions, please reply to this message or contact the journal office at `plosone@plos.org`. When you're ready to submit your revision, log on to `https://www.editorialmanager.com/pone/` and select the 'Submissions Needing Revision' folder to locate your manuscript file.

Please include the following items when submitting your revised manuscript:

- *A rebuttal letter that responds to each point raised by the academic editor and reviewer(s). You should upload this letter as a separate file labeled 'Response to Reviewers'.*
- *A marked-up copy of your manuscript that highlights changes made to the original version. You should upload this as a separate file labeled 'Revised Manuscript with Track Changes'.*
- *An unmarked version of your revised paper without tracked changes. You should upload this as a separate file labeled 'Manuscript'.*

If you would like to make changes to your financial disclosure, please include your updated

statement in your cover letter. Guidelines for resubmitting your figure files are available below the reviewer comments at the end of this letter.

If applicable, we recommend that you deposit your laboratory protocols in `protocols.io` *to enhance the reproducibility of your results. Protocols.io assigns your protocol its own identifier (DOI) so that it can be cited independently in the future. For instructions see:* `http://journals.plos.org/plosone/s/submission-guidelines#loc-laboratory-protocols`

We look forward to receiving your revised manuscript.

Kind regards,

Ivan Kryven
Academic Editor
PLOS ONE

Response:

Thank you very much for the revision.

We have modified the paper and supplementary material attending to the comments and suggestions raised by Reviewer 1. A point-by-point response is provided here. The modifications have been marked in red color in the new version of the paper.

We have condensed the description of the steps of our approach by providing details in the Supplementary Material. We have also included more discussion concerning the example and extensions, condensing all these extensions. Additionally, a mimic example based on the real data set with its R code, data, and instructions to replicate it is now included as part of the manuscript.

It is important to note that since the last revision, we have added funding for this research, and some lines have to be included in the manuscript concerning this matter. We have added them next to the Acknowledgment section.

Reviewers' comments:

Reviewer's Responses to Questions

Comments to the Author

---

1. If the authors have adequately addressed your comments raised in a previous round of review and you feel that this manuscript is now acceptable for publication, you may indicate that here to bypass the "Comments to the Author" section, enter your conflict of interest statement in the "Confidential to Editor" section, and submit your "Accept" recommendation.

Reviewer #1: (No Response)

---

2. Is the manuscript technically sound, and do the data support the conclusions?

The manuscript must describe a technically sound piece of scientific research with data that supports the conclusions. Experiments must have been conducted rigorously, with appropriate controls, replication, and sample sizes. The conclusions must be drawn appropriately based on the data presented.

Reviewer #1: (No Response)

---

3. Has the statistical analysis been performed appropriately and rigorously?

Reviewer #1: (No Response)

Have the authors made all data underlying the findings in their manuscript fully available?

The PLOS Data policy requires authors to make all data underlying the findings described in their manuscript fully available without restriction, with rare exception (please refer to the Data Availability Statement in the manuscript PDF file). The data should be provided as part of the manuscript or its supporting information, or deposited to a public repository. For example, in addition to summary statistics, the data points behind means, medians and variance measures should be available. If there are restrictions on publicly sharing data–e.g. participant privacy or use of data from a third party–those must be specified.

Reviewer #1: (No Response)

5. Is the manuscript presented in an intelligible fashion and written in standard English?

PLOS ONE does not copyedit accepted manuscripts, so the language in submitted articles must be clear, correct, and unambiguous. Any typographical or grammatical errors should be corrected at revision, so please note any specific errors here.

Reviewer #1: (No Response)

6. Review Comments to the Author

Reviewer #1:

The revision is a substantial improvement because the example now plays a more central role throughout, making it easier to assess the relevance of the proposed method.

Response

Thank you very much for the revision. We really appreciate your time in reviewing the paper.

We have modified the paper attending to the comments and suggestions raised by you. A point-by-point response is provided here. The modifications have been marked in red color in the new version of the paper.

(1) *The proposed method itself is rather straightforward. If we log-transform data and fit a linear mixed model assuming normality, then the analysis implies that the untransformed data have a log-normal distribution. The naive back-transformation of means from the log-scale to the original scale does now provide unbiased estimators of the expected value on the original scale. This is all well known, and it is also known that the mean on the original scale is simple* $exp(\mu) * (exp(\sigma^2/2))$. *All the authors are, therefore, proposing is that we should multiply the naive estimator* $exp(\hat{\mu})$ *with the improved estimator* $exp(\hat{\mu}) * (exp(\hat{\sigma}^2/2))$. *This works straightforwardly for a linear mixed model, we just need to work out what the marginal variance is in each case.*

It is not clear why the authors are using so much matrix algebra to make this very simple point. The matrix algebra is clearly unnecessary, even if generalizing this to other models. Thus, I see a lot of scope form simplification. Certainly, the matrix algebra does not make things easier in any way as asserted in L202. How could things be possibly any easier than in the scalar multiplication shown in eq. (9)? And that's all it takes!

Response

It is correct that the idea behind our result is a correction involving the expected value of the exponential of a random normal distribution u, $\mathrm{E}[\exp(u)] = \exp(\mu) * (\exp(\sigma^2/2))$, involving $\exp(\sigma^2/2)$, where μ and σ^2 corresponds to the mean and variance of u. We

obtained different types of estimators, but the one with the best performance is based on the same idea used to "estimate" the random effects (which are random variables and not parameters) in a linear mixed model (LMM), in which the best linear unbiased predictors (BLUP) are used. The BLUP for the random effects corresponds to the expected value associated with the distribution of these effects conditional to the distribution of the response in the full sample (the Y's). Thus, for the proposed correction term associated with the random effect, we require the expected value of the exponential of a random variable corresponding to a conditional distribution. Hence, we need the variance associated with such conditional distribution. In contrast, the variance of the marginal distribution of the random effects is simply σ_{γ}^2, obtaining expression (5), which was also analyzed, but with inferior results. In particular, for the random intercept model (RIM), we need the conditional distribution of a univariate normal random variable, the random effect, conditional to a multivariate normal distribution, the log-transformed response. Once obtaining this variance, the procedure to correct for the random effect is as usual, using the exponential value of the variance divided by two. However, as we are obtaining the conditional distribution of a univariate conditional to a multivariate distribution, it is necessary to perform linear algebra to obtain a closed, non-matricial (scalar), reduced, and easy to use expression for the variance associated with the conditional distribution (which is still normal) as presented in equation (13) (previous version, (10) in new version). In the end, we obtain the estimator in equation (14) (previous version, (11) in new version). The estimator in equation (9) is simpler, because instead of using $\mathrm{E}[\exp(u)]$ for u a normal (conditional) distribution associated with the random effects, we are using $\exp[\mathrm{E}[u]]$, which is easier to obtain since $\mathrm{E}[u]$ simply corresponds to the BLUP for the random effects provided by any software ($\hat{\gamma}_i$); however, according to the Jensen inequality this is a lower bound of the true value as discussed in lines 196-198. Consequently, in some instances, particularly under heteroscedasticity, we observed similar estimations using (9) and (14) (previous version, (11) in new version).

When we use a LMM in general (with any number of random effects), the process is analogous, but we have a multivariate normal distributional conditioned to another multivariate normal distribution, and for each individual in a cluster, we require the distribution associated with the linear combination associated with the random effects.

We have rewritten and simplified the part in which we present the correction term based on the expected values of the conditional distribution (lines 200-219), so that the process is now clearer and shorter. We have moved all algebra to Proposition 1 in the Supplementary

material, but we have written it in such a way that this material is self-contained, and not a particular case of the general LMM. This process has greatly reduced this part in the manuscript. However, some material that was included here concerning the LMM in general had to be incorporated to the section corresponding to LMM (lines 416-432) and the Supplementary material was modified and corrected to consider this new focus.

(2) *In the same vein, the authors' proposal to use simulation in case other transformations than the logarithm are used is fine, though not new either. Moreover, this can be said in a single sentence. Then three pages the authors are devoting to this can be shortened substantially, if not removed altogether. Instead, they could focus on analytical results that are available for the power-normal distribution:*

Freeman, J., and R. Modarres. 2006. Inverse Box–Cox: The powernormal distribution. Stat. Probab. Lett 76:764–772. `doi:10.1016/j.spl.2005.10.036`

Response

Thank you for providing us with this reference. It is important to notice that, as we have mentioned in the previous answer, that one type of estimator is obtained without conditioning to the response and another by conditioning to it, expression (5) *vs.* expression (11) (revised manuscript) for a RIM, having expression (11) a better performance. The results concerning Freeman and Modearres (2006), we think can be used for estimations similar to the ones in equation (5). For an expression similar to (11) we would need to condition a power normal distribution to a normal distribution, the latter corresponding to the transformed data in which the LMM is fitted. Hence, we think that for the type of estimator similar to (11), the simulation method should be used. We have applied the results in Freeman and Modearres (2006) and simplified the part corresponding to the simulation.

It is important to notice that in the last section, what corresponded to other type of transformations besides of logarithm, was from line 456 to line 489 (previous version) and not all section. The first part corresponded to LMM and a logarithmic transformation, i.e. including any number of random effects, using once again in this part an estimation based on a conditional distribution for the random effects. The other type of estimators, without conditioning, are a particular case of the results with the power-normal distribution, which perhaps are the ones the reviewer is considering. Thus, three type of generaliza-

tions are now included in the manuscript: 1) Logarithmic transformation in LMM, with corrections based on the conditional distribution (lines 413 to 448), which have a close formula, 2) Box-Cox transformations with corrections in LMM based on a distribution without conditioning based on Freeman and Modearres (2006) (lines 458 to 468), which also have a close formula, and 3) Any invertible function in LMM, based on the conditional distribution and simulations (lines 469 to 478). We tried to simplify the explanation of all three aspects, for instance moving the part concerning the variance of the conditional distribution associated with the LMM to the Supplementary section (Proposition 2). To simplify even more the part corresponding to LMM, we could also erase the paragraph corresponding to an example considering two random effects, from line 449 to line 457.

(3) *The authors make a bit point about heteroscedasticity, but the form of heteroscedasticity is very restrictive (L145): they assume that the variance is σ^2/w_i, where w_i are known weights. This is, of course, the most favourable case, but most of the time such weights are unknown. This begs two questions: (i) what to do if heteroscadasticity is of a different form and (ii) what are the weights in the central real example? They mention in L32 what the weights could be, but what about their example?*

Response

The form in which we present heteroscedasticity is the usual one, in the sense that it is assumed to have fixed known weights, σ^2_{ij}, as used for instance in weighted least squares estimators. We used the expression $\sigma^2 w_{ij}^{-1}$ instead of the general form σ^2_{ij} since it is the way it is presented in software, for instance in R, in which a constant variance is always included and additionally we include the weights. However, the results in the general form σ^2_{ij} are followed from what we obtained simply replacing $\sigma^2 w_{ij}^{-1}$ for σ^2_{ij} in all the expressions. Other variance structures, for instance in R varPower() or VarExp(), are simply functions depending on certain parameters (and a constant term σ^2) concerning variables which seem to be associated with heteroscedasticity. As far as we know, once these parameters are estimated, the weights could be assumed as known, by simply calculating these values for each individual using the estimated parameters. Hence, we do not see any problem in using these variance structures in the same way as the weights we have included. We have added a sentence concerning this matter in the discussion (lines 509-515).

Maybe the question is how to obtain or approximate these weights. As mentioned in the

manuscript, one form is by using the Elbers and collaborators method (ELL method); which depends on fitting two models, one marginal fitted by OLS and a second one corresponding to transformations of the residuals obtained in the first model. For the second model, we should include variables that we know have an impact over the heteroscedasticity, though they can be the same as in the first model. Some other options are using as weights the size associated with each unit or some of the explanatory variables. In our example, we stated that homoscedasticity was used (lines 360-362); however, we used the ELL method with the same explanatory variables for both the alpha and beta model, as well as other types of weightening, for instance simply using the squared residuals and the cluster size. The model using the weights through the ELL method provided slightly better predictions, with a squared root of the mean squared error (MSE) of 6804.41 *vs.* 6829 when the weights were not used (homoscedasticity). However, in terms of the normality assumption, a better performance was obtained assuming the same variance, considering for the normality qq-plot the weights as explained in the previous revision (graph under the ELL method shown below). For the other types of weightening, we obtained larger values for the root of the MSE than those obtained with the ELL method or homoscedasticity (for instance 6871.6 when the squared residuals were used). Hence, we decided to present only the homoscedastic case for our example, but now we have included some of these matters in the example (lines 394-397). Since this happened for our data set, we decided that the simulations would be the part in our manuscript in which illustrate the heteroscedastic case and now we have included a simulation based on the real data set (lines 398-410) as explained in the next answer.

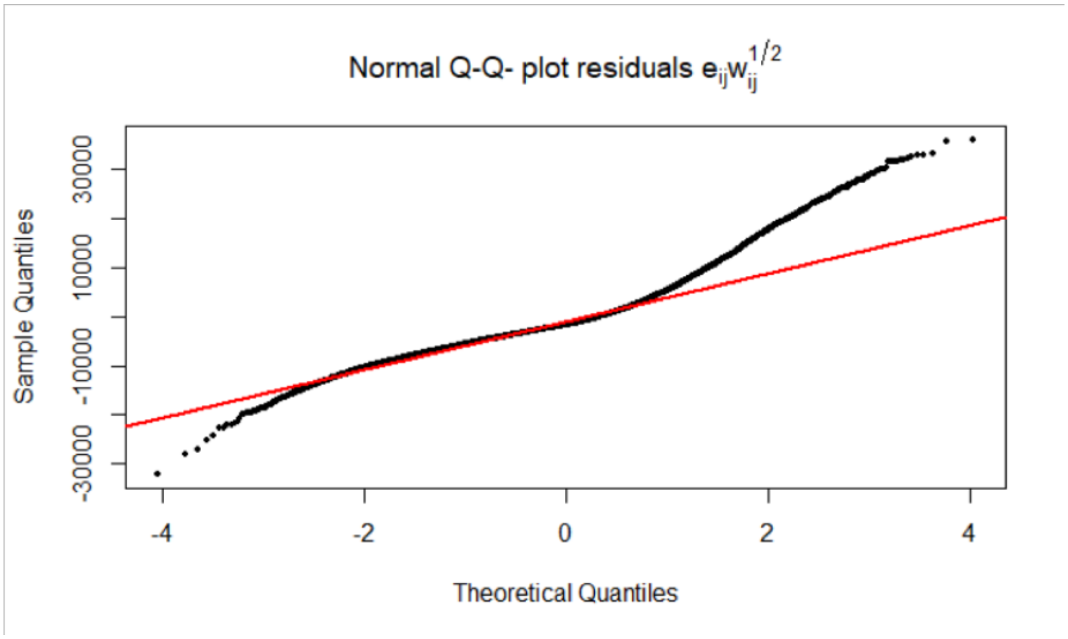

(4) *As regards the simulation, it would be very useful to explain how the parameter setting is modelled on the real example. As it stands, it seems like the settings studied fall from the skies, which is not very convincing. In particular, coming back to the weights, what is the rationale for the simulation of the weights in L283-284? What do the weights represent in the real-life application the simulation is hopefully representing here?*

Response

The goal of the simulation we presented was to evaluate the way our method worked in an ideal setting in which the weights are known and necessary, not necessarily emulating the real data. In that simulation we wanted to see how the method worked after varying the clusters size, considering the balanced and unbalanced case, and to evaluate our methods, even considering other models used for similar data. Of course, the weights could have been tried to be estimated in each sample, but then, we would be evaluating the technique for the estimation for the weights instead of the correction. However, we have included now simulations using mimic data, that is, data simulated from the real data information. In this example, a sample of the real dataset is obtained, where the explanatory variables are used to simulate the response variable several times. The weights were selected in two cases: (1) proportional to one of the explanatory variables, and (2) to the unit size, as it is common in real-life applications. These simulations were added to the manuscript

and a detailed description of the simulation process in the Supplementary material to avoid having a considerable increase in the manuscript length. The source R code, data, and instructions that allow to replicate the analysis for the mimic simulation example are available as Supplementary Material.

Finally, ss mentioned in the previous answer, in our data set a best performance was obtained with a same variance used for all the individuals, though we tried to include the weights in different forms. It is also important to notice that heteroscedasticity is part of the models we define and the corrections we use, but its analysis is not the main objective of our manuscript, and as shown in our example, a model without them could work for some data.

Further remarks:

• L141: Something wrong here with the representation of the normal as $N(\sigma^2, \sigma^2)$

Corrected

• L154: y => and

Corrected

---

7. PLOS authors have the option to publish the peer review history of their article (what does this mean?). If published, this will include your full peer review and any attached files.

If you choose "no", your identity will remain anonymous but your review may still be made public.

Do you want your identity to be public for this peer review? For information about this choice, including consent withdrawal, please see our Privacy Policy.

Reviewer #1: No

[NOTE: If reviewer comments were submitted as an attachment file, they will be attached to this email and accessible via the submission site. Please log into your account, locate the manuscript record, and check for the action link "View Attachments". If this link does not appear, there are no attachment files.]

While revising your submission, please upload your figure files to the Preflight Analysis and Conversion Engine (PACE) digital diagnostic tool, `https://pacev2.apexcovantage.com/`. *PACE helps ensure that figures meet PLOS requirements. To use PACE, you must first register as a user. Registration is free. Then, login and navigate to the UPLOAD tab, where you will find detailed instructions on how to use the tool. If you encounter any issues or have any questions when using PACE, please email PLOS at* `figures@plos.org`. *Please note that Supporting Information files do not need this step.*
