# Supplementary material for: Random intercept and linear mixed models including heteroscedasticity in a logarithmic scale: Correction terms and prediction in the original scale
Source: PLoS One. 2021 Apr 14;16(4):e0249910. doi: 10.1371/journal.pone.0249910 (PMC8046211; doi:10.1371/journal.pone.0249910)
Supplement: Attachment — Submitted filename: Response to Reviewers_02032021.pdf [file pone.0249910.s009.pdf]

PONE-D-20-31434R2

PLOS ONE

Random intercept and linear mixed models including heteroscedasticity in a logarithmic scale: correction terms and prediction in the original scale

Ricardo Ramírez-Aldana, Lizbeth Naranjo

Academic Editor

Dear Dr. Ramírez-Aldana,

Thank you for resubmitting your manuscript to PLOS ONE. Reviewer 1 has noticed that one of their comments was not understood as intended, please see below. We therefore invite you to respond to this comment by incorporating the necessary changes to the manuscript.

Please submit your revised manuscript by Mar 26 2021 11:59PM. If you will need more time than this to complete your revisions, please reply to this message or contact the journal office at `plosone@plos.org`*. When you're ready to submit your revision, log on to* `https://www.editorialmanager.com/pone/` *and select the 'Submissions Needing Revision' folder to locate your manuscript file.*

Please include the following items when submitting your revised manuscript:

- *A rebuttal letter that responds to each point raised by the academic editor and reviewer(s). You should upload this letter as a separate file labeled 'Response to Reviewers'.*
- *A marked-up copy of your manuscript that highlights changes made to the original version. You should upload this as a separate file labeled 'Revised Manuscript with Track Changes'.*
- *An unmarked version of your revised paper without tracked changes. You should upload this as a separate file labeled 'Manuscript'.*

If you would like to make changes to your financial disclosure, please include your updated

statement in your cover letter. Guidelines for resubmitting your figure files are available below the reviewer comments at the end of this letter.

If applicable, we recommend that you deposit your laboratory protocols in protocols.io to enhance the reproducibility of your results. Protocols.io assigns your protocol its own identifier (DOI) so that it can be cited independently in the future. For instructions see: `http://journals.plos.org/plosone/s/submission-guidelines#loc-laboratory-protocols`

We look forward to receiving your revised manuscript.

Kind regards,

Ivan Kryven
Academic Editor
PLOS ONE

Response:

Thank you very much for the revision.

We have modified the paper attending to the comment raised by Reviewer 1. A point-by-point response is provided here. The modifications have been marked in red color in the new version of the paper.

Reviewers' comments:

Reviewer's Responses to Questions

Comments to the Author

---

1. 1. If the authors have adequately addressed your comments raised in a previous round of review and you feel that this manuscript is now acceptable for publication, you may indicate that here to bypass the "Comments to the Author" section, enter your conflict of interest statement in the "Confidential to Editor" section, and submit your "Accept" recommendation.

Reviewer #1: (No Response)

---

2. Is the manuscript technically sound, and do the data support the conclusions?

The manuscript must describe a technically sound piece of scientific research with data that supports the conclusions. Experiments must have been conducted rigorously, with appropriate controls, replication, and sample sizes. The conclusions must be drawn appropriately based on the data presented.

Reviewer #1: (No Response)

---

3. Has the statistical analysis been performed appropriately and rigorously?

Reviewer #1: (No Response)

---

4. Have the authors made all data underlying the findings in their manuscript fully available?

The PLOS Data policy requires authors to make all data underlying the findings described in their manuscript fully available without restriction, with rare exception (please refer to the Data Availability Statement in the manuscript PDF file). The data should be provided as part of the manuscript or its supporting information, or deposited to a public repository. For example, in addition to summary statistics, the data points behind means, medians and variance measures should be available. If there are restrictions on publicly sharing data–e.g. participant privacy or use of data from a third party–those must be specified.

Reviewer #1: (No Response)

---

5. Is the manuscript presented in an intelligible fashion and written in standard English?

PLOS ONE does not copyedit accepted manuscripts, so the language in submitted articles must be clear, correct, and unambiguous. Any typographical or grammatical errors should be corrected at revision, so please note any specific errors here.

Reviewer #1: (No Response)

---

6. Review Comments to the Author

Please use the space provided to explain your answers to the questions above. You may also include additional comments for the author, including concerns about dual publication, research ethics, or publication ethics. (Please upload your review as an attachment if it exceeds 20,000 characters)

---

6. Review Comments to the Author

Reviewer #1:

The authors acknowledge that in case of heteroscedasticity the weights are typically unknown and hence need to be estimated. They further mention two options in R to estimate such functions and note that these involve parameters. It may be added that common forms of heteroscedasticity may also involve correlation, as is the case, e.g., with repeated measures data. The authors further response is based on this assertion: "As far as we know, once these parameters are estimated, the weights could be assumed as known, by simply calculating these values for each individual using the estimated parameters." This is true as far as the point estimates for fixed and random effects are concerned, and only when residual errors are independently distributed, but it is incorrect as far as the inference is concerned. Adjustments are needed both regarding the standard errors and the degrees of freedom. See the papers by Kenward and Roger (1997 and 2009 in Biometrics and CSDA).

I would like the authors to re-consider this point, and I am happy to take another look once that's done.

Response

Thank you very much for the revision. We really appreciate your time in reviewing the paper. We have modified the paper attending to the comment raised by you. A point-by-point response is provided here. The modifications have been marked in red color in the new version of the paper.

It is important to clarify that when heteroscedasticity is referred, it is assumed that the variance and covariance structure associated with the error can have different values in the diagonal, but always zero outside, which under a normality distribution assumption corresponds to assuming independence. This structure is also assumed in the models with different variance forms available in R that we mentioned in the previous answer. Hence, we did not assume any covariance structure for the error, as for instance, one involving time. However, a generalization of our results for any covariance structure is possible using the results we present for the general form of the LMM in line 419, since in the general framework, a matrix Σ_i with any correlation structure for each cluster can be used, instead of just a diagonal matrix as we assumed in line 431. All calculations we presented there are the same when any matrix Σ_i is used, instead of just a diagonal matrix. This implies that the correction presented in equation (17) applies for any covariance structure (only

modifying the third term corresponding to the error term and taking care when inferences are involved).

We think that in our previous answer we were not clear in that the model including the heteroscedasticity structure (including parameters for this part) should be first fitted, and then the estimated parameters should be used in the formulas for the correction terms, which depends on both the fixed and random effects parameters. In fact, the process to fit a LMM (both with ML or REML estimation processes) depends first on estimating all the terms concerning the variance and covariance structure for both the error and random effects (included in matrices Σ_i and D, respectively, according to the notation we used for the corresponding variance and covariance matrices) in the profile or marginal log-likelihood function, and once estimating them, they are used in a generalized least squares (GLS) estimator for the fixed effects. Thus, following the same idea, the correction would use the estimated variance and covariance structure and corresponding estimated parameters. However, as mentioned by the reviewer, any inference would require to consider that the correlation structure is being estimated, and that the degrees of freedom of the asymptotic distribution should be corrected or, as in the references provided by the reviewer [1] and [2], corrections should be applied when small sample sizes or non-linear covariance structures are involved. Even in the best scenario, it has been shown that the variance and covariance associated with the estimated parameters, and consequently their standard errors, are biased, and thus the variance and covariance structure should be as close as possible to the real one. As far as we know, this process of correction of the degrees of freedom is performed in R when a correlation structure is included, thus the inference, particularly for the fixed effects should be correct, at least when small sample sizes are not involved. However, it is important to notice that we do not use the standard error of the estimated parameters in our correction.

We now indicate that the model should be first fitted, including the possible heteroscedasticity structure (which could depend on some parameters), and that any inference should be performed with care being aware that the degrees of freedom are corrected or correction terms applied for the inference (as in the references provided by the reviewer). After that, they can use the estimated parameters in the process for predicting the response in the original scale.

References

[1] Kenward MG. Small sample inference for fixed effects from restricted maximum likelihood. Biometrics. 1997;53:983–997.

[2] Kenward MG, Roger JH. An improved approximation to the precision of fixed effects from restricted maximum likelihood. Computational Statistics and Data Analysis. 2009;53:2583–2595.
